# SNP Assay Development for Linkage Map Construction, Anchoring Whole-Genome Sequence, and Other Genetic and Genomic Applications in Common Bean

**DOI:** 10.1534/g3.115.020594

**Published:** 2015-08-28

**Authors:** Qijian Song, Gaofeng Jia, David L. Hyten, Jerry Jenkins, Eun-Young Hwang, Steven G. Schroeder, Juan M. Osorno, Jeremy Schmutz, Scott A. Jackson, Phillip E. McClean, Perry B. Cregan

**Affiliations:** * USDA-ARS, Soybean Genomics and Improvement Lab, Beltsville, Maryland 20705; † HudsonAlpha Institute for Biotechnology, Huntsville, Alabama 35806; ‡ Department of Plant Science and Landscape Architecture, University of Maryland, College Park, Maryland 20742; § USDA-ARS, Bovine Functional Genomics Laboratory, Animal and Natural Resources Institute, Beltsville, Maryland 20705; ** Department of Plant Sciences, North Dakota State University, Fargo, North Dakota 58102; †† Joint Genome Institute, 2800 Mitchell Drive, Walnut Creek, California 94598; ‡‡ Center for Applied Genetic Technologies, University of Georgia, Athens, Georgia 30602

**Keywords:** *Phaseolus vulgaris*, SNP, molecular markers, BARCBean6K BeadChip, linkage map

## Abstract

A total of 992,682 single-nucleotide polymorphisms (SNPs) was identified as ideal for Illumina Infinium II BeadChip design after sequencing a diverse set of 17 common bean (*Phaseolus vulgaris* L) varieties with the aid of next-generation sequencing technology. From these, two BeadChips each with >5000 SNPs were designed. The BARCBean6K_1 BeadChip was selected for the purpose of optimizing polymorphism among market classes and, when possible, SNPs were targeted to sequence scaffolds in the *Phaseolus vulgaris* 14× genome assembly with sequence lengths >10 kb. The BARCBean6K_2 BeadChip was designed with the objective of anchoring additional scaffolds and to facilitate orientation of large scaffolds. Analysis of 267 F_2_ plants from a cross of varieties Stampede × Red Hawk with the two BeadChips resulted in linkage maps with a total of 7040 markers including 7015 SNPs. With the linkage map, a total of 432.3 Mb of sequence from 2766 scaffolds was anchored to create the *Phaseolus vulgaris* v1.0 assembly, which accounted for approximately 89% of the 487 Mb of available sequence scaffolds of the *Phaseolus vulgaris* v0.9 assembly. A core set of 6000 SNPs (BARCBean6K_3 BeadChip) with high genotyping quality and polymorphism was selected based on the genotyping of 365 dry bean and 134 snap bean accessions with the BARCBean6K_1 and BARCBean6K_2 BeadChips. The BARCBean6K_3 BeadChip is a useful tool for genetics and genomics research and it is widely used by breeders and geneticists in the United States and abroad.

DNA molecular markers, especially simple sequence repeats (SSRs) and single-nucleotide polymorphisms (SNPs), are used widely for the construction of linkage maps, mapping of quantitative trait loci (QTL), map-based gene cloning, marker-assisted selection, exploration of population diversity, etc. in all major crops including maize (Sharopova *et al.* 2002; Xiang *et al.* 2001), soybean (Chen *et al.* 2007; Molnar *et al.* 2003; Song *et al.* 2004, 2010), wheat (Anderson *et al.* 2001; Roder *et al.* 1998; Song *et al.* 2005), rice (Brondani *et al.* 2002; McCouch *et al.* 1997), and cotton (Karaca *et al.* 2002; Wang *et al.* 2006). In common bean (*Phaseolus vulgaris* L), primer sets were developed for approximately 121,000 SSR loci derived from expressed sequence tags and genomic DNA and were reported at http://phaseolusgenes.bioinformatics.ucdavis.edu/. Of these, about 500 SSR markers were mapped genetically with different mapping populations (Blair *et al.* 2009, 2011, 2012a,b; Grisi *et al.* 2007; Hanai *et al.* 2010; Rodriguez-Suarez *et al.* 2007; Yu *et al.* 2000; Yuste-Lisbona *et al.* 2012). Recently, a set of 2687 high-quality indel markers were identified, and some of the markers were genetically mapped (Moghaddam *et al.* 2014). SNPs are the most abundant form of DNA polymorphism in eukaryotic genomes (Brookes 1999) and are suitable for development of high-throughput, easy-to-automate genotyping methods (Ding and Jin 2009; Yoon *et al.* 2007). In common bean, a set of 239 SNPs was first reported by comparing sequences from coding and noncoding regions and genomic DNA of a set of 10 diverse genotypes (Gaitan-Solis *et al.* 2008). Subsequently, 118 SNP markers were identified from expressed sequence tags and were genetically mapped in the DOR364 × G19833 recombinant inbred line population (Galeano *et al.* 2009) and 1580 SNPs were identified by sequence analysis of introns and three-prime untranslated regions of 550 genes in the common bean genotypes, JaloEEP558 and BAT93 (McConnell *et al.* 2010). Most recently, with the advent of next-generation sequencing technologies, approximately 4000 SNPs were reported by comparing alignments of sequences from different genotypes (Blair *et al.* 2013; Hyten *et al.* 2010). However, as the whole genome sequence of common bean was not available when this previous research was conducted, information regarding the genome position of the SNPs, the specificity of the SNP flanking sequences, and the distance between adjacent SNPs was not available.

The Joint Genome Institute, Department of Energy has released the first chromosome scale version of *Phaseolus vulgaris* (v1.0) (http://www.phytozome.net/) (Schmutz *et al.* 2014). Before this version, two *de novo* assemblies (14× and v0.9) were produced. The 14× scaffold assembly contained 470.1 Mb of sequence. It consisted of 10,037 scaffolds with sequence length >2 kb but <2.5 Mb, and of these, 22 and 4525 scaffolds were longer than 1 Mb and 10 kb, respectively. Among the 5512 scaffolds with sequence length less than 10 kb, 4284 scaffolds were shorter than 5 kb. Anchoring a large portion of the completed sequence to create 11 pseudomolecules was essential.

The objectives of this research were to efficiently identify large numbers of SNPs with high polymorphism within and between common bean market classes that were different types of the *Phaseolus vulgaris* species originally domesticated in the Mexico and South America, to develop high-throughput Illumina Infinium BeadChips, to create genetic linkage maps, and to anchor or orient large scaffolds of the common bean DNA sequence assembly.

## Materials and Methods

### Selection of a diverse set of common bean varieties from different market classes for SNP discovery

To identify a diverse set of genotypes for sequencing, a total of 192 common bean genotypes consisting of varieties from nine market classes representing most common bean diversity were genotyped with a GoldenGate assay containing 1307 SNPs (Hyten *et al.* 2010). After monomorphic SNPs or SNPs with a high rate of missing data or heterozygotes were eliminated, a total of 1159 SNPs were used to generate a neighbor joining tree of the 192 genotypes (Supporting Information, Figure S1) and to count the maximum number of SNPs detectable by a combination of a predefined number of genotypes in each market classes. A set of genotypes within each market class that could jointly detect the maximum number of SNPs and that were well distributed in different phylogeny clusters was selected. By the use of this analysis, a total of 15 diverse varieties, including Red Hawk, Fiero, Cal Early, Kardinal, Lark, Cornell 49242, T-39, UI 906, Laker, C-20, Michelite, Buckskin, Stampede, Sierra, and Matterhorn, were identified for high-throughput sequencing. The 15 selected varieties included varieties from nine of the major common bean market classes and captured >98% of the 1159 SNPs that were polymorphic among 192 diverse genotypes assayed with the GoldenGate assay (Hyten *et al.* 2010). In addition, BAT93 and Jalo EEP558, which are the parents of a widely used *P. vulgaris* mapping population (Freyre *et al.* 1998; Grisi *et al.* 2007; McClean *et al.* 2002; Nodari *et al.* 1993), also were included.

### Sequencing a panel of 17 common bean varieties with the Illumina Genome Analyzer IIx

DNA of the 17 common bean varieties was extracted from seeds and digested with dsDNA fragmentase (Linnarsson 2010). Index sequences 6 bp in length were ligated to the resulting fragments and 350–800 bp fragments were size selected on a 1.0% agarose gel, purified, and prepared for Illumina GAIIx sequencing (Illumina Inc, San Diego, CA). The Off-line Basecaller Software (v1.7; Illumina Inc) was used for base calling and CASAVA (v 1.7; Illumina Inc) was used for mapping the paired-end reads to the common bean 14× genome sequence assembly generated by the Joint Genome Institute and to identify SNPs. Parameters of a minimum of three reads at the SNP position and a SNP quality score >10 were used for SNP allele calling.

### Design of Illumina BeadChips BARCBean6K_1 and BARCBean6K_2 for SNP analysis

The design of BARCBean6K_1 and BARCBean6K_2 chips followed the procedures previously described (Song *et al.* 2013), *i.e.*, A/T or G/C SNPS were eliminated, as were SNPs with N’s in the 60 nt of flanking sequence, SNPs residing within 25 nt of another SNP, and SNPs for which the 25-nt flanking sequences were not unique in the genome. Sequences flanking putative SNPs were evaluated with the use of Illumina’s Assay Design Tool (http://www.illumina.com/applications/microarrays/microarray-software/array-probe-design.html), and SNPs with design score <0.6 were excluded. The BARCBean6K_1 SNPs were selected for the purpose of optimizing polymorphism among the various common bean market classes and, when possible, SNPs were targeted to scaffolds in the *Phaseolus vulgaris* 14× assembly with sequence lengths greater than 10 kb. The 14× assembly was the only assembly available at the time when the BARCBean6K_1 BeadChip was designed. When the *Phaseolus vulgaris* v0.9 assembly became available, another BeadChip, the BARCBean6K_2, was designed based on the sequence of the v0.9 assembly. The v0.9 assembly consists of 10,132 scaffolds with sequence lengths ranging from 1 kb to 2.5 Mb; however, only 76 of the scaffolds were greater than 1 Mb. SNPs were selected with the objective of anchoring additional sequence scaffolds and to facilitate the orientation of large scaffolds via the selection of additional SNPs in the case of scaffolds with only one marker polymorphic in the Stampede × Red Hawk mapping population based on the BARCBean6K_1 analysis. When more than one SNP was chosen from the same scaffold, SNPs at the distal ends of the scaffold were preferred if it was possible. The 60-bp sequences flanking the SNPs as well as the SNP positions along the linkage groups were used to anchor the scaffolds of the v0.9 assembly and to position SNPs on the *Phaseolus vulgaris* 1.0 assembly (http://www.phytozome.net/).

### Validation of the BARCBean6K_1 and BARCBean6K_2 Illumina Infinium BeadChips

To determine the rate of successful SNP calls, the 17 varieties used for sequencing and SNP discovery were genotyped with the two BeadChips. Procedures of SNP genotyping and determination of SNP alleles using the Infinium II assay were previously described (Song *et al.* 2013, 2015).

### Construction of genetic linkage maps

A mapping population of 267 F_2_ plants from the cross of common bean varieties Stampede and Red Hawk developed by Drs. Phil McClean and Juan Osorno at North Dakota State University was genotyped with the BARCBean6K_1 and BARCBean6K_2 BeadChips. In addition, 484 SNP loci that were genotyped with the Illumina GoldenGate assay (Hyten *et al.* 2010) and 25 framework markers that had been mapped previously in the Stampede × Red Hawk F_2_ population also were included for linkage map construction. The 25 framework markers mapped to the 11 *Phaseolus vulgaris* linkage groups and thereby allowed the identification of the 11 linkage groups. Linkage maps were constructed with the software JoinMap 4.0 (Van Ooijen 2006).

### Selection of a core set of 6000 SNPs (BARCBean6K_3)

To provide a tool for breeders and geneticists to identify genes or QTL associated with their traits of interest, 6000 SNPs were selected from the successful markers on the BARCBean6K_1 and BARCBean6K_2 BeadChips for inclusion in the BARCBean6K_3 BeadChip. Only SNPs that had a minor allele frequency (MAF) >0.05 and with missing or ambiguous allele calls <10% among 365 dry bean and 134 snap bean accessions (Table S1) from the Association Mapping Population of the BeanCAP (http://www.beancap.org/) were selected for inclusion in the BARCBean6K_3 BeadChip. In addition, SNPs that were polymorphic in more than one common bean market class, that were located on different scaffolds, or that were located in different bins of the linkage maps of the Stampede × Red Hawk population were preferentially selected when possible, so that the number of SNPs, especially in the heterochromatic regions which had very low recombination rates, was greatly reduced. This procedure of SNP selection, which was based on the genetic distance/physical distance ratio in the heterochromatic and euchromatic regions, was previously reported in soybean (Song *et al.* 2013).

### Data availability

All data related to SNPs contained in the BARCBean6K_1, BARCBean6K_2 and BARCBean6k_3 Beadchips, as well as the Stampede × Red Hawk F2 genetic map are available in Table S2, Table S3, Table S4, and Table S5.

## Results

### Sequencing and SNP discovery

A total of 286.7 million reads totaling 32,970 Mbp of sequence was generated from the DNA of the 17 common bean varieties. Sequence coverage varied from 1.6× to 5.2× based on an estimated whole-genome sequence length of 637 Mbp (Arumuganathan and Earle 1991) and between 57.0 and 72.5% of the paired-end reads from the 17 varieties uniquely aligned to the 14× *P. vulgaris* assembly. Using the CASAVA 1.7 software, we identified 1,857,237 SNPs based on alignment to the 14× common bean genome sequence, which is equal to approximately 3 SNPs per kilobase genome-wide. Among these SNPs, a total of 21,449, 7954, 49,655, 36,310, and 213,087 SNPs were polymorphic among varieties within the Light Red Kidney, Dark Red Kidney, Black, Navy, and Pinto market classes, respectively.

### Development of BeadChips BARCBean6K_1 and 6 BARCBean6K_2

After applying the various SNP filters, the remaining 992,682 SNPs were candidates for design of the BARCBean6K_1 and BARCBean6K_2 BeadChips (Table 1). The 6000 SNPs included in the BARCBean6K_1 were chosen from 1636 scaffolds with a total sequence length of 314.8 Mb. In those cases when more than one SNP was selected from the same scaffold, the distance between the SNPs was always >5.5 kb. All of the 6000 SNPs were polymorphic within at least one market class, 4326 and 210 SNPs were polymorphic within two, and more than two of the nine market classes, respectively. MAF among the 17 varieties was ≥0.13 for all of the SNPs based upon the initial sequence alignment to the 14× assembly. (Table S2).

**Table 1:** Number of SNPs retained after filtering steps during the analysis of 32,720 Mbp of 121-bp Illumina paired-end sequence of genomic DNA of 17 common bean genotypes

Filters	Number of SNPs Retained
Polymorphic among 17 common bean varieties	1,857,237
Elimination of SNPs residing within 25 nt of another SNP	1,232,064
Elimination of SNPs with A/T or G/C alleles	993,237
Elimination of SNPs with N in the 60-nt regions flanking the SNP	992,682

SNP, single-nucleotide polymorphism.

For the development of the BARCBean6K_2 assay, SNPs that were used in the BARCBean6K_1 BeadChip or that were monomorphic between the Stampede and Red Hawk parents of the F_2_ mapping population were removed from the dataset of 992,682 SNPs. Of the 6000 SNPs selected for inclusion in the BARCBean6K_2 BeadChip, a total of 4063 SNPs were selected from 2381 scaffolds that had not been targeted by SNPs in the BARCBean6K_1 BeadChip. A total of 1336 SNPs were from the 599 scaffolds that contained SNPs in the BARCBean6K_1 BeadChip but were not polymorphic between the Stampede and Red Hawk parents. In addition, 429 SNPs were from the 194 scaffolds with only one polymorphic SNP in the BARCBean6K_1 BeadChip and 173 SNPs were selected from large scaffolds that contained a small number of SNPs polymorphic between Stampede and Red Hawk in the BARCBean6K_1 BeadChip (Table S3).

### SNP validation using the set of varieties that were used for SNP discovery

A total of 5399 (90%) of the 6000 SNPs were included as bead types in the BARCBean6K_1 bead pool and 5514 (92%) of the 6000 SNPs were included as bead types in the BARCBean6K_2 bead pool, of which, 5320 (98.5%) and 5308 (96.3%) of the SNPs were validated, respectively. Approximately 95% of the SNPs in both assays had a MAF greater than 10% among the 17 varieties (Table S2 and Table S3).

### Construction of the common bean linkage map

The 267 F_2_ plants of the Stampede × Red Hawk population were genotyped with the BARCBean6K_1 and BARCBean6K_2 BeadChips. After elimination of SNPs with missing data >10% or loci with significant segregation distortion from a 1:2:1 ratio as measured by χ^2^ at the 1% significant level, 6531 SNPs were retained for linkage analysis. Analysis of 7040 markers including 25 framework markers and 484 previously mapped SNPs produced a genetic map comprising 11 consensus linkage groups that spanned 1042.2 cM of Kosambi map distance (Table S4). The average number of markers mapped per linkage group was 640 ranging from 225 to 979 (Table 2).

**Table 2:** Number of SNPs mapped to each chromosome and linkage group lengths

Chromosome	Total Number of Markers	Number of Newly Mapped SNPs	Number of Framework Markers	Number of Previously Mapped SNPs [Hyten *et al*. 2010]	Map Length, cM
Chr01	834	760	2	72	84.0
Chr02	568	518	2	48	127.6
Chr03	588	536	2	50	116.9
Chr04	757	703	2	52	94.0
Chr05	697	666	2	29	90.8
Chr06	229	199	2	28	70.8
Chr07	225	192	3	30	107.8
Chr08	863	822	3	38	114.0
Chr09	492	449	2	41	94.6
Chr10	808	763	3	42	60.2
Chr11	979	923	2	54	81.5
Total	7040	6531	25	484	1042.2

SNP, single-nucleotide polymorphism.

### Anchoring the scaffolds to build the *Phaseolus vulgaris* 1.0 assembly

The 60-bp sequences flanking the 7015 SNPs as well as the SNP positions along the linkage groups were used to build the *Phaseolus vulgaris* 1.0 assembly. With the SNP-based linkage maps, a total of 432.3 Mb of sequence from 2766 scaffolds was anchored which accounted for approximately 89% of the 486.9 Mb of total sequence from 10,132 scaffolds of the *Phaseolus vulgaris* v0.9 assembly (Table S4). In addition, 65 of the 66 scaffolds with sequence length >1 Mb and 83 of the 130 scaffolds with sequence length between 50 kb and 1 Mb were oriented on the linkage map based upon the detection of genetic recombination between the two or more markers which were positioned on these scaffolds.

### Position of SNPs on the *Phaseolus vulgaris* 1.0 assembly

The 60-bp SNP flanking sequences were used to align the SNPs to the *Phaseolus vulgaris* v1.0 assembly. Of the 12,000 SNPs originally identified for inclusion in the two BeadChips, 11,837 were uniquely positioned on chromosomes 1−11, and 51 aligned to 41 unanchored scaffolds of the *Phaseolus vulgaris* v1.0 assembly (Table S2 and Table S3). The number of the 12,000 SNPs on the 11 chromosomes ranged from 750 to 1404 (Table 3).

**Table 3:** The SNPs included as bead types in the BARCBean6K_1, BARCBean6K_2, and BARCBean6K_3 BeadChips and the predicted common bean chromosome on which they were located

Chromosome	Number of SNPs Included in the BARCBean6K_1 and BARCBean6K_2 BeadChips	Number of SNPs Included in the BARCBean6K_3 BeadChip
Chr01	1209	504
Chr02	1082	579
Chr03	795	361
Chr04	1234	534
Chr05	1255	574
Chr06	550	311
Chr07	1092	458
Chr08	1324	572
Chr09	750	419
Chr10	1142	451
Chr11	1404	573
SNPs present in unanchored scaffolds	51	62
Total	11,837	5398

SNP, single-nucleotide polymorphism.

### Polymorphism of a core set of 6000 SNPs (BARCBean6K_3 BeadChip) in the diverse group of dry bean and snap beans

Based on the analysis of a total of 365 dry bean and 134 snap bean accessions genotyped with BARCBean6K_1 and BARCBean6K_2, a total of 10,039 SNPs remained after eliminating SNPs with MAF <5% and missing or ambiguous allele calls >10%. Among the 6000 SNPs selected for BARCBean6K_3 from the 10,039 candidate SNPs, a total of 5398 were included in the bead pool. Of these a total of 3159, 4494, and 4316 SNPs were polymorphic in the races Nueva Granada, Mesoamerican, and Durango, respectively, and 4939 SNPs were polymorphic within the snap beans. Among the 5316 SNPs polymorphic within at least one race of dry bean, a total of 718, 2543, and 2055 SNPs were polymorphic within one, two, or three races, respectively. This included 2583 SNPs polymorphic within both the Middle American and Andean gene pools and 2733 polymorphic in either the Middle American or Andean gene pools. The average MAF of the 5398 SNPs in the bead pool based on 499 BeanCAP genotypes was 0.32 (Table S5).

## Discussion

With the advent of high-throughput sequencing technology and advanced SNP identification pipelines, a total of 1.8 million SNPs in the common bean genome were discovered based on a panel of 17 diverse varieties of common bean from different market classes, *i.e.*, approximately three SNPs per kilobase in the common bean genome. The 1.8 million SNPs were identified from 8766 scaffolds of the 14× *P. vulgaris* assembly. Among 4525 scaffolds with sequence length >10 kb, 4501 contained at least one SNP. Genotyping of the 17 varieties with the BARCBean6K_1 and the BARCBean6K_2 assays indicated that >97% of the SNPs were validated. Thus, the SNPs identified in this research are a valuable source of SNPs for additional BeadChip or other SNP assay development.

The first two BeadChips developed in this study are the first Illumina Infinium BeadChips in common bean and are useful tools for genetic and genomic research in this important plant species. The BARCBean6K_1 BeadChip was designed to characterize genetic diversity among and within common bean market classes, and 100% of the SNPs included in the BeadChip were polymorphic within one market class, 76% were polymorphic within at least two market classes and, in addition, all SNPs had a MAF >0.13 and average MAF of 0.33 among the 17 varieties used in the SNP discovery. Although SNPs included in the BARCBean6K_2 were chosen to capture the polymorphism between the Stampede and Red Hawk mapping population parents, the SNPs were highly polymorphic among accessions from various market classes. Of the 5398 SNPs included in the BARCBean6K_3 beadchip, a total of 1396, 1024, 2087, 1893, and 3388 SNPs were polymorphic among varieties within the Light Red Kidney, Dark Red Kidney, Black, Navy, and Pinto market classes, respectively. The chip will be useful even in a population of Light Red Kidney varieties, which are known to have very limited genetic diversity.

Genetic linkage maps are the basic tool to localize QTL or genes in the genome. In common bean, most of the genetic linkage maps were based on SSR or other types of molecular markers. Yu *et al.* (2000) developed a linkage map consisting of 232 markers including 15 SSRs. Blair *et al.* (2003) constructed a map with 246 markers including 78 SSRs, 48 restriction fragment-length polymorphism, 102 random amplified polymorphic DNAs, and 18 amplified fragment length polymorphisms. Grisi *et al.* (2007) created a map with 231 markers including 113 SSRs and 118 other molecular markers. Galeano *et al.* (2009) mapped 288 markers including 118 markers with single-strand conformation polymorphisms. Cordoba *et al.* (2010) integrated 99 SSRs into the common bean genetic map. In addition, a linkage map of 275 markers consisting of 199 gene-based SNPs was constructed by McConnell *et al.* (2010). Hanai *et al.* (2010) developed a linkage map containing 413 DNA markers including SSRs and AFLPs. Our research provided a linkage map with 7040 markers including 7015 SNPs. To date this is the linkage map with the highest density of molecular markers in common bean. In addition, the linkage map was generated in a large population (>260 F_2_ plants) and resulted in a map of high resolution and accuracy. It is anticipated that the linkage map and the SNPs it contains will facilitate QTL mapping and marker assisted selection in common bean.

The high-density linkage map created in this study resulted in the anchoring of 2766 scaffolds that cover 89% of the length of the genome of the *Phaseolus vulgaris* v0.9 assembly and facilitated the building of the *Phaseolus vulgaris* 1.0 assembly. Of the 7366 unanchored scaffolds, 5691 had a sequence length of less than 5 kb and 735 scaffolds ranged from 5 to 10 kb in length. Together, these 735 scaffolds accounted for 36% of the total unanchored sequence length. Most of the sequences on the 13 unanchored scaffolds with sequence length between 20 and 40 kb were highly repetitive. Sequences flanking the SNPs on these scaffolds were generally not specific or were otherwise inappropriate for Illumina Infinium assay design. To properly orient scaffolds, at least two markers with large intervals between them were chosen for each large scaffold in the hope that recombination between such markers would allow their proper orientation. As indicated, 65 of the 66 scaffolds with sequence length >1 Mb and 83 of the 130 scaffolds with sequence length between 50 kb and 1 Mb were oriented on the linkage map in this manner. In those cases when scaffolds could not be oriented, an analysis based on synteny to soybean was used to assist in their orientation.

Many researchers believe that the level of diversity within common bean races is limited (Kwak and Gepts 2009; McClean and Lee 2007; Santalla *et al.* 2010), because each race of common bean is characterized by distinctive morphological, agroecological, and molecular phenotypes (McClean and Lee 2007). Thus, it is essential to develop markers that are polymorphic within each race to be useful for genetic research and diversity analysis. At least, 85% and 38% of the SNPs on the BARCBean6K_3 BeadChip were polymorphic within two and three common bean races, respectively. The BARCBean6K_3 BeadChip has been applied to genotype recombinant inbred lines of mapping populations and germplasm by breeders and geneticists in the United States and Brazil. The resulting datasets will assist in the identification of QTL/genes, the application of genome-wide association analysis of important traits, identification of signatures of selection and evolution, and the determination of genetic diversity and genome regions associated with common bean domestication.

## 

## Supplementary Material

Supporting Information
