# Supplementary material for: SNP Assay Development for Linkage Map Construction, Anchoring Whole-Genome Sequence, and Other Genetic and Genomic Applications in Common Bean
Source: G3 (Bethesda). 2015 Aug 28;5(11):2285–90. doi: 10.1534/g3.115.020594 (PMC4632048; doi:10.1534/g3.115.020594)
Supplement: Supporting Information [file supp_5_11_2285__index.html]

SNP Assay Development for Linkage Map Construction, Anchoring Whole-Genome Sequence, and Other Genetic and Genomic Applications in Common Bean — Supporting Information 

# SNP Assay Development for Linkage Map Construction, Anchoring Whole-Genome Sequence, and Other Genetic and Genomic Applications in Common Bean

## Supporting Information for Song *et al.*, 2015

**Files in this Data Supplement:**

- Supporting Information - Figure S1 and descriptions of Tables S1-S5 (PDF, 832 KB)
- Figure S1 - Neighbor joining tree derived from the analysis of 192 common bean varieties with 1,159 SNPs (Hyten *et al.* 2010) and identification of 15 (encircled in red) of the 17 cultivars used for whole genome sequence analysis for SNP discovery. (PDF, 693 KB)
- Table S1 - Market class and race of common bean accessions genotyped with the BARCBean6K\_1 and BARCBean6K\_2 Illumina BeadChips. (.xlsx, 25 KB)
- Table S2 - BARCBean6K\_1 SNPs and analysis. (.xlsx, 877 KB)
- Table S3 - BARCBean6K\_2 SNPs and analysis. (.xlsx, 859 KB)
- Table S4 - Linkage group and linkage position (cM) of markers in the Stampede x Red Hawk (SR) F2 linkage map and alignment of the SNP flanking sequence to the v0.9 *Phaseolus vulgaris* genome assembly. (.xlsx, 373 KB)
- Table S5 - The BARCBean6K\_3 SNPs and analysis. (.xlsx, 1 MB)
