# Supplementary material for: SNP Assay Development for Linkage Map Construction, Anchoring Whole-Genome Sequence, and Other Genetic and Genomic Applications in Common Bean
Source: G3 (Bethesda). 2015 Aug 28;5(11):2285–90. doi: 10.1534/g3.115.020594 (PMC4632048; doi:10.1534/g3.115.020594)
Supplement: Supporting Information [file supp_g3.115.020594_020594SI.pdf]

G3·Genes | Genomes | Genetics

SNP assay development for linkage map construction, anchoring whole genome sequence and other genetic and genomic applications in common bean

Qijian Song*, Gaofeng Jia[*], David L. Hyten*[1], Jerry Jenkins[†], Eun-Young Hwang[‡], Steven G. Schroeder[§], Juan M. Osorno[**], Jeremy Schmutz[†,††], Scott A. Jackson[‡‡], Phillip E. McClean[**], Perry B. Cregan[*]

[*]USDA-ARS, Soybean Genomics and Improvement Lab, Beltsville, MD 20705

[†] HudsonAlpha Institute for Biotechnology, Huntsville, Alabama 35806, USA

[‡]Department of Plant Science and Landscape Architecture, University of Maryland, College Park, MD 20742

[§]USDA-ARS, Bovine Functional Genomics Laboratory, Animal and Natural Resources Institute, Beltsville, MD, 20705, USA

[**]Department of Plant Sciences, North Dakota State University, Fargo, ND 58102, USA

[††]Joint Genome Institute, 2800 Mitchell Drive, Walnut Creek, California 94598, USA.

[‡‡]Center for Applied Genetic Technologies, University of Georgia, Athens, Georgia 30602, USA.

[1] **Present address:** Dep. of Agronomy and Horticulture, 322 Keim Hall, University of Nebraska, Lincoln, NE 68583, USA

Correspondence author: Qijian Song, Soybean Genomics and Improvement Laboratory, Beltsville Agricultural Research Center – West, USDA, ARS, Beltsville, MD 20705-2350. Tel: 301-504-5723, e-mail: Qijian.Song@ars.usda.gov

DOI: 10.1534/g3.115.020594

Tables S1-S5

Available for download as Excel files at www.g3journal.org/lookup/suppl/doi:10.1534/g3.115.020594/-/DC1

Table S1. Market class and race of common bean accessions genotyped with the BARCBean6K_1 and BARCBean6K_2 Illumina BeadChips.

Table S2. BARCBean6K_1 SNPs and analysis: SNP ID, NCBI submitted SNP ID number, SNP alleles, Chromosome and SNP position in the v1.0 *P. vulgaris* assembly, 60nt of SNP flanking sequence, Minor Allele Frequency (MAF) of the SNP in the sequence analysis of the 17 cultivars, Illumina SNP Design Score, Inclusion of SNP in the BARCBean6K_1 beadpool (Y/N), Polymorphism of the SNP in the BARCBean6K_1 analysis, MAF >0.05 in the analysis of the 499 BeanCAP lines (Y/N) and Ambiguous allele calls in the BeanCAP line analysis <10% (Y/N)

Table S3. BARCBean6K_2 SNPs and analysis: SNP ID, NCBI submitted SNP ID number, SNP alleles, Chromosome and SNP position in the v1.0 *P. vulgaris* assembly, 60nt of SNP flanking sequence, Minor Allele Frequency (MAF) of the SNP in the sequence analysis of the 17 cultivars, Illumina SNP Design Score, Inclusion of SNP in the BARCBean6K_2 beadpool (Y/N), Polymorphism of the SNP in the BARCBean6K_2 analysis, MAF >0.05 in the analysis of the 499 BeanCAP lines (Y/N) and Ambiguous allele calls in the BeanCAP line analysis <10% (Y/N).

Table S4. Linkage group and linkage position (cM) of markers in the Stampede x Red Hawk (SR) F_2 linkage map and alignment of the SNP flanking sequence to the v0.9 *Phaseolus vulgaris* genome assembly

Table S5. The BARCBean6K_3 SNPs and analysis: SNP ID; NCBI ssID; Included in the BARCBean6K_3 Beadpool (Y/N); Chromosome and SNP position in the v1.0 *P. vulgaris* assembly; Minor Allele Frequency (MAF) of the SNP in the sequence analysis of the 499 diverse BeanCAP common bean genotypes; % of BeanCAP lines with missing or ambiguous allele calls; Polymorphism in the common bean races Nueva Granada, Mesoamerican, and Durango and in the snap bean genotypes; Polymorphism within the Middle American and Andean genepools; Number of dry bean races with polymorphism; Linkage group and cM position in the Stampede x Red Hawk mapping population; SNP location in a gene (Y/N); Position in gene if applicable; Source of the SNP either BARCBean6K_1 or BARCBean6K_2 BeadChip and the 60 nt of SNP flanking sequence.

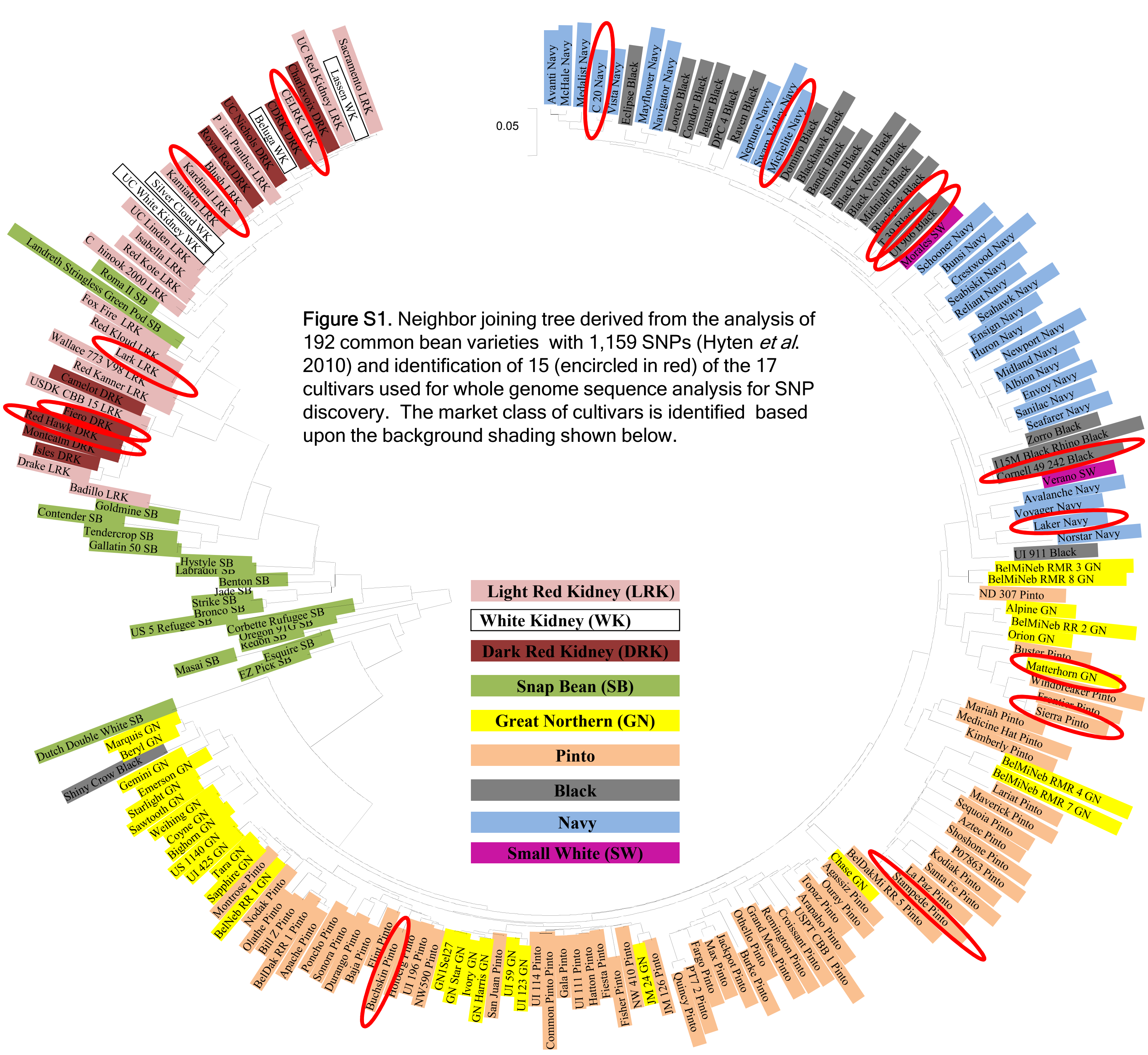

Figure S1. Neighbor joining tree derived from the analysis of 192 common bean varieties with 1,159 SNPs (Hyten *et al.* 2010) and identification of 15 (encircled in red) of the 17 cultivars used for whole genome sequence analysis for SNP discovery. The market class of cultivars is identified based upon the background shading shown below.
