# Supplementary figures and images for: SNP Assay Development for Linkage Map Construction, Anchoring Whole-Genome Sequence, and Other Genetic and Genomic Applications in Common Bean
Source: G3 (Bethesda). 2015 Aug 28;5(11):2285–90. doi: 10.1534/g3.115.020594 (PMC4632048; doi:10.1534/g3.115.020594)

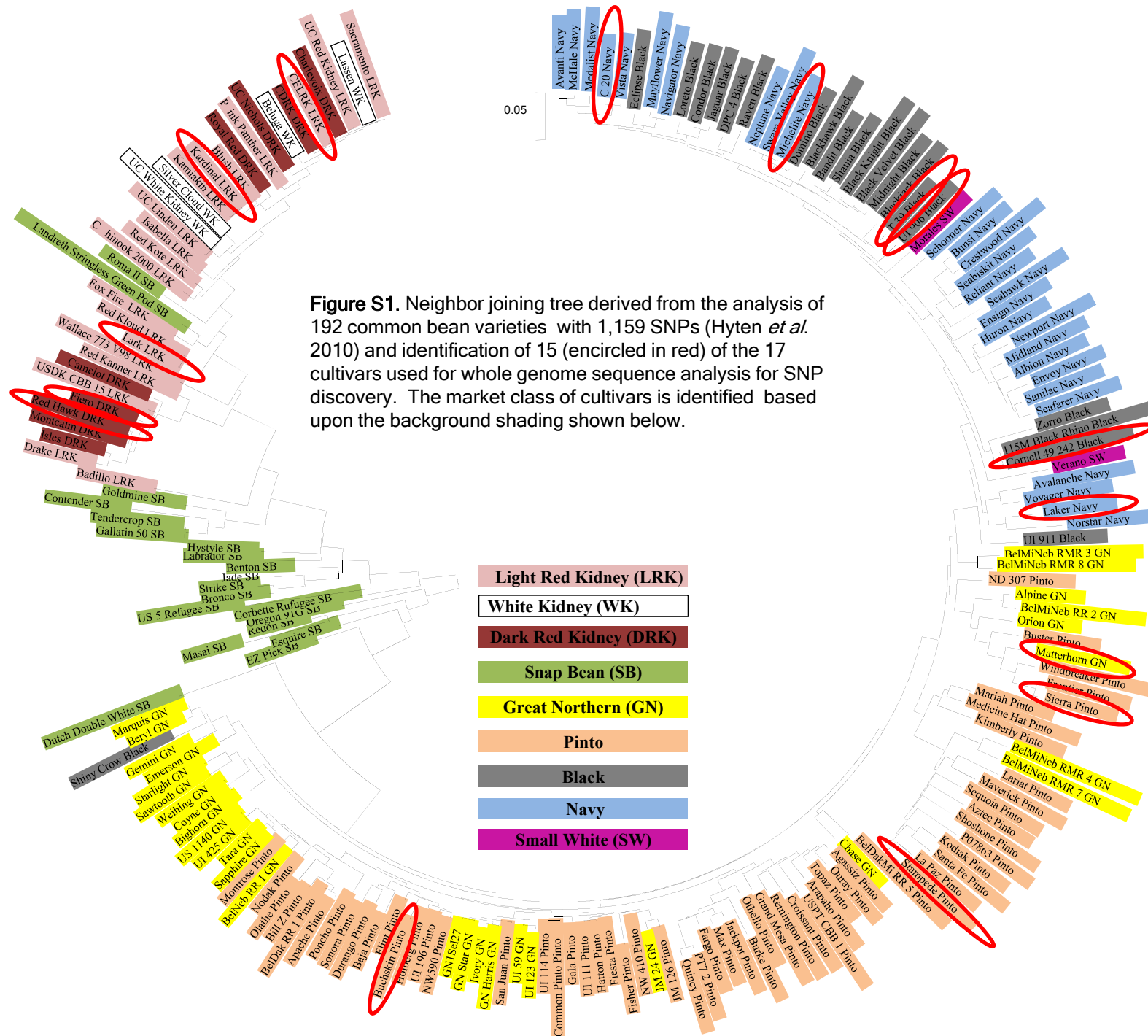

Supplement: Supporting Information [file supp_g3.115.020594_FigureS1.pdf]
